# What are the predictors of malignancy in inflammatory bowel disease?

**DOI:** 10.55730/1300-0144.6036

**Published:** 2025-04-24

**Authors:** Zülal İSTEMİHAN, Bilger ÇAVUŞ, Ali Emre BARDAK, Ayşe Merve KIRKOĞLU, Cansu KIZILTAŞ, Rüveyda SİLAY, İbrahim Volkan ŞENKAL, Ziya İMANOV, Kenan NURİYEV, Aynure RÜSTEMZADE, Sezen GENÇ, Mine GÜLLÜOĞLU, Aslı ÇİFCİBAŞI ÖRMECİ, Kadir DEMİR, Fatih BEŞIŞIK, Sabahattin KAYMAKOĞLU, Filiz AKYÜZ

**Affiliations:** 1 Division of Gastroenterohepatology, Department of Internal Medicine, İstanbul Faculty of Medicine, İstanbul University, İstanbul, Turkiye; 2 Department of Internal Medicine, İstanbul Faculty of Medicine, İstanbul University, İstanbul, Turkiye; 3 Department of Pathology, İstanbul Faculty of Medicine, İstanbul University, İstanbul, Turkiye

**Keywords:** Biologics, immunomodulators, inflammatory bowel disease, malignancy

## Abstract

**Background/aim:**

This study aims to investigate the prevalence of malignancy in patients with inflammatory bowel disease (IBD) followed up in a tertiary reference center.

**Materials and methods:**

IBD patients with at least 6 months of follow-up from the gastroenterology clinic between 2000 and 2022 were evaluated retrospectively in a tertiary center. Patient information was obtained from the patient registration system.

**Results:**

There were 697 patients in the study, 320 (45.9%) of these were female. The mean age of the patients at IBD diagnosis was 33.4 ± 13.1 years. The mean follow-up time was 93.1 ± 64.8 (median 84, IQR (25–75) (36–144)) months. IBD types were as; 315 (45.2%) had ulcerative colitis, and 382 (54.8%) had Crohn’s disease. Before the diagnosis of IBD, 10 patients (1.4%) had a history of malignancy. The prevalence of malignancy after the diagnosis of IBD was 13 (1.9%). There was a relationship between malignancy and older age, higher BMI, and female sex in survival analysis (p < 0.05). There was no correlation with disease type and malignancy (p = 0.820). When the patients who developed malignancy in IBD were compared in terms of the immunomodulators and biological agents used by the patients who did not develop malignancy, there was no statistical difference between the two groups (p > 0.05).

**Conclusion:**

Older age, higher BMI, and female sex were found to be at risk for the development of malignancy in IBD, although no relationship was found with the use of immunomodulators, biological agents, or disease type.

## 1. Introduction

Inflammatory bowel diseases (IBD) are chronic idiopathic bowel diseases that progress with remissions and relapses. Chronic inflammation is an important risk factor for gastrointestinal malignancies in the long term [1]. The most common cancers in the background of IBD are gastrointestinal system malignancies, and colorectal cancers are the most common among them [2]. Nowadays, both gastrointestinal and non-gastrointestinal system malignancies are increasing, especially with the increase in immunosuppression and prolongation of the duration of immunosuppression with the developing treatments [3]. However, as in all malignancies, the development of malignancy in patients with IBD is multifactorial, and information about the criteria associated with it is limited.

This study aims to investigate the prevalence of malignancy in patients with IBD followed up in a tertiary reference center.

## 2. Materials and methods

Our study is a single-center and retrospective cohort study. There are 697 patients diagnosed with IBD and at least six months of follow-up in our IBD clinic between 2000–2022. The demographic information of the patients and the information about their diseases were examined in detail. The relationship between the demographic characteristics of the patients, comorbidities, disease characteristics, and the development of malignancy was evaluated.

Characteristics of IBD were divided into ulcerative colitis (UC), and Crohn’s disease (CD). UC patients were classified according to the involvement pattern as pancolitis, left colon involvement, and distal colon involvement. CD patients were classified as having ileal involvement, ileocolonic involvement, colonic involvement, and upper gastrointestinal tract involvement in addition to others, according to the pattern of involvement.

The patients were also investigated in terms of their history of malignancy before the diagnosis of IBD, and it was examined whether de-novo or recurrence of malignancy developed in these patients during their IBD treatment and follow-up. De novo malignancy or de novo cancer is the first occurrence of cancer in the body. Recurrence of malignancy is the development of the same malignancy again after a certain disease-free period in a patient who has previously received treatment for malignancy and has been cured.

Patients who developed malignancies after the diagnosis of IBD were evaluated in terms of the most common malignancies. Patients who developed malignancy after the diagnosis of IBD were specifically examined in terms of the treatments used for the treatment of IBD before and after the malignancy diagnosis. Patients were evaluated for immunomodulatory drugs (methotrexate, azathioprine, or 6-mercaptopurine) and biological agents (anti-TNFs, anti-integrin, or anti-IL12/23) use. After the development of malignancy, IBD treatment management was reviewed.

There were 10 patients with a history of malignancy before the diagnosis of IBD. These were patients who were cured of their malignancies before the diagnosis of IBD. It was observed that these patients did not need immunosuppressive therapy during the follow-up of IBD treatment.

Approval for this study was obtained from the Ethics Committee of Istanbul University Faculty of Medicine on 09.09.2022. The protocol number is 2022/1488. All the applied procedures complied with the ethical standards of the Human Testing Committee of our institution and the Helsinki Declaration. Due to the study’s retrospective nature, the requirement for written informed consent was waived, and approval was received from the ethics committee of the Istanbul Faculty of Medicine.

### 2.1. Statistical analysis

Descriptive statistics for continuous variables in the study; mean, median, and standard deviation; and categorical variables were expressed as numbers (n) and percentages (%). An independent T-test was performed to compare measurements according to categorical groups. The chi-square test was calculated to determine the relationships between categorical variables. Survival curves were estimated using the Kaplan–Meier method, and compared using the log-rank test. Multivariate analysis was performed based on the Cox proportional hazards regression model. Statistical significance level (a) was taken as 5% in the calculations, and SPSS (IBM SPSS for Windows, ver.25) statistical package program was used for analysis.

## 3. Results

### 3.1. Demographic characteristics of all patients

There were a total of 696 patients in the study. Of these patients, 320 (45.9%) were female. The mean age of the patients was 33.4 ± 13.1 years at diagnosis of IBD. The mean disease age or follow-up duration of the patients was 93.1 ± 64.8 (6–264) months. The patients’ mean body mass index (BMI) was 23.2 ± 4.5 kg/m^2^. Of the patients, 315 (45.2%) had UC, and 382 (54.8%) had CD. UC patients were older, overweight, and had longer disease duration than CD patients (p = 0.002, p = 0.006, and p = 0.020, respectively) (Table 1).

498 (71.4%) patients were using immunomodulatory drugs, 246 (35.3%) patients were using biological agents, and 224 (32.1%) patients were using both immunomodulatory drugs and biological agents. 178 (25.5%) patients were not using immunomodulatory drugs or biological agents. The mean duration of immunosuppressive drug use was 91.6 ± 62.5 months. When drugs are examined separately, the mean immunomodulator drug use was 92.8 ± 62.4 months, the mean biological agent use was 80.6 ± 57.4 months, and the mean both immunomodulatory drugs and biological agents was 82.1 ± 57.2 months.

In survival analyses, no association was observed between the use of immunomodulators, biological agents, or their combined use and the development of malignancy (p = 0.595, p = 0.191, and p = 0.252, respectively).

Seronegative spondyloarthropathy was present in 39 (5.6%) patients. 21 (3%) had diabetes mellitus, and 27 (3.9%) had hypertension. 131 (18.8%) patients were smokers.

Before the diagnosis of IBD 10 (1.4%) patients had a history of malignancy. Four patients had breast cancer, one patient had basal cell carcinoma, one patient had bladder cancer, one patient had renal cell carcinoma, one patient had ovarian cancer, one patient had endometrial cancer, and one patient had primary splenic lymphoma. None of these patients developed malignancy again during their IBD process.

When CD patients were evaluated individually, 203 (53.1%) of these patients were male and 179 (46.9%) were female. The mean age at diagnosis of CD patients was 32 ± 12.5 years, the mean disease age was 87.9 ± 60.5 (median 72, IQR (25–75) (36132)) months, and the mean BMI was 22.5 ± 4.6 kg/m^2^. 150 (39.3%) of Crohn’s patients had ileocolonic involvement. Of the CD patients, 215 (56.3%) had inflammatory, 60 (15.7%) had stenosis and 107 (28) had fistulizing disease. 27 (7.1%) patients had seronegative spondyloarthropathy accompanying CD. 99 (25.9%) of the CD patients were smokers. Of the CD patients, 324 (84.8%) were using immunomodulators, 198 (51.8%) were using biological agents, and 179 (46.9%) were using a combination of biological agents and immunomodulators. Forty of them (10.5%) were not using immunomodulators or biological agents. 5 (1.3%) of Crohn’s patients had a history of malignancy before CD diagnosis. 6 (1.6%) of CD patients developed malignancy after IBD diagnosis.

When UC patients were evaluated individually, 174 (55.2%) of the patients were male and 141 (44.8%) were female. The mean age at diagnosis of UC patients was 35.1 ± 13.8 years, the mean disease age was 99.5 ± 69.1 (median 96, IQR (25–75) (36–156)) months, and the mean BMI was 24 ± 4.2 kg/m^2^. Thirty-two (10.2%) were smokers. One hundred and eighty-two (57.8%) were pancolitis, 74 (23.5%) were diseases with distal involvement, and 59 (18.7%) were diseases with left colon involvement. Five patients (1.6%) were accompanied by seronegative spondyloarthropathy. One hundred and seventy-four (55.2%) were using immunomodulatory drugs, 48 patients (15.2%) were using biological agents, and 45 patients (14.3%) were using immunomodulators and biological agents combined. 138 patients (43.8%) were not using immunomodulatory drugs or biological agents. Thirty-two (10.2%) were smokers. Five (1.6%) patients had a history of malignancy before the diagnosis of UK. Malignancy developed after UC diagnosis in 7 (2.2%) patients.

### 3.2. Patients who developed malignancy and their characteristics

The number of patients who developed malignancy after the diagnosis of IBD was 13 (1.86%). The malignancy that developed in all of these patients was de novo cancer. We did not have any cases of cancer recurrence. The mean age of the patients who developed malignancy was 42.8 ± 12.2 years at IBD diagnosis, the mean disease age was 116.1 ± 76.8 (median 144, IQR (25–75) (36–174)) months, and the mean BMI was 28.4 ± 5.3 kg/m^2^. None of these patients had a history of malignancy before the diagnosis of IBD.

Of the patients who developed malignancy after the diagnosis of IBD, 10 (76.9%) were females, and 3 (23.1%) were males. CD was present in 6 patients (46.2%) and UC in 7 patients (53.8%). None of the patients were smokers. 3 (42.9%) patients with UC had pancolitis, 3 (42.9%) had left colon involvement, and 1 had distal colon involvement. 2 (33.3%) of those with CD had ileocolonic involvement, and others (n = 4, 66.7%) had ileal involvement. 3 (50%) of the CD patients had inflammatory, 2 (33.3%) had fistulising, and 1 had stenosing disease.

Only two of the patients who developed malignancy were using biologic agents, one of which was adalimumab and the other was infliximab.

The most common malignancy was colorectal adenocarcinoma, in 5 patients (38.4%). Other malignancies were Kaposi’s sarcoma, pancreatic cancer, diffuse large B-cell lymphoma (DLBCL), cervical cancer, neuroendocrine cancer, spindle cell cancer, mycosis fungoides, and breast cancer, in one patient each. All patients who developed malignancy were in clinical and endoscopic remission before the development of malignancy.

The relationship between sex, disease type, age, BMI, disease age, medications, comorbidities, and development of malignancy in IBD was examined with survival analysis. In the survival analysis, there was no correlation between the type of disease and the development of malignancy (p = 0.820). In our study, a significant relationship was found between the female sex and the development of malignancy, but this value is statistically borderline (p = 0.046) (Table 2). However, when men and women were compared in terms of age, BMI, follow-up duration, and follow-up duration of immunosuppressive drug use, no difference was seen between them (p = 0.793, p = 0.235, p = 0.071, and p = 0.640, respectively; the mean age of women was 48.1 ± 15.1 years, the mean age of men was 48.4 ± 14.1 years; the mean BMI of women is 22.8 ± 5.1 kg/m^2^, the mean BMI of men was 23.4 ± 3.9 kg/m^2^; the mean follow-up duration for women was 97.2 ± 67.7 months, the mean follow-up duration for men was 89.7 ± 62 months; the mean follow-up duration of immunosuppressive drug use for women was 93 ± 63.8 months, the mean follow-up duration of immunosuppressive drug use for men was 90.4 ± 61.6 months). However, when the group that developed malignancy was examined, colorectal cancers were significantly more common in men (p = 0.012; colorectal cancer rate was 100% in men and 20% in women).

No relationship was found between the use of immunomodulatory drugs or biological agents and the development of malignancy (p = 0.595, and p = 0.191, respectively), and also the mean duration of immunosuppressive drug use was not different between the groups (91.5 ± 62.3 months in those who did not develop malignancy, 97 ± 77.2 months in those who developed malignancy; p = 0.788). Diabetes mellitus, hypertension, seronegative spondyloarthropathy, and smoking were not related to malignancy development (p = 0.682, p = 0.146, p = 0.831, and p = 0.106, respectively).

The area under the curve (AUC) of the diagnosis age for malignancy development after IBD diagnosis was 0.711 [95% confidence interval (CI) = 0.587–0.836, p = 0.009]. At an optimal cut-off of 40 years old, the diagnosis age had a sensitivity of 77% and a specificity of 71.6% for malignancy development after IBD diagnosis. The mean age at diagnosis of patients who developed malignancy after IBD diagnosis was higher than those who did not develop malignancy (p = 0.010) (the mean age at diagnosis of IBD patients with malignancy was 42.8 ± 12.2 years, and 33.2 ± 13.1 years without malignancy) (Table 2).

The area under the curve (AUC) of the BMI for malignancy development after IBD diagnosis was 0.794 [95% confidence interval (CI) = 0.615–0.973, p = 0.008]. At an optimal cut-off of 25 kg/m^2^, the BMI had a sensitivity of 71.4% and a specificity of 75.8% for malignancy development after IBD diagnosis. The mean age at diagnosis of patients who developed malignancy after IBD diagnosis was higher than those who did not develop malignancy (p = 0.002) (the mean BMI at diagnosis of IBD patients with malignancy was 28.4 ± 5.3 kg/m^2^, and 23 ± 4.4 kg/m^2^ without malignancy) (Table 2).

The relationship between malignancy development and disease age in IBD was examined. When examined according to periods of 96 and 120 months, interestingly, patients with disease durations of less than 96 and 120 months were found to be associated with the development of malignancy compared to those with longer disease durations (p = 0.002 and p = 0.026, respectively).

The association between the female sex, being diagnosed with IBD over the age of 40, BMI ≥ 25 kg/m^2^, and the development of malignancy was examined in multivariate Cox regression analysis. The relationship between sex, age and malignancy could not be obtained in multivariate analysis, but the relationship between overweight and malignancy was also shown in multivariate analysis (HR = 1.634; 95% CI = 0.361 – 7.403; p = 0.524, HR = 1.706; 95% CI = 0.262–11.133; p = 0.577, and HR = 13.991; 95% CI = 1.307–149.749; p = 0.029; respectively) (Table 3).

IBD treatments were organized as follows. The treatment of these patients for IBD was arranged as 5-aminosalicylic acid (5-ASA) only in 11 patients. One patient with colorectal cancer who underwent total proctocolectomy did not receive treatment for IBD after surgery. The patient who used infliximab before breast cancer developed was treated with vedolizumab. The characteristics of these 13 patients are described in Table 4.

## 4. Discussion

Inflammatory bowel diseases are chronic, idiopathic, relapsing, and remission bowel diseases, the etiology of which is still unclear, and possible environmental and genetic factors play a role. The prevalence of IBD is increasing in the world, and today, with new treatment options, survival in IBD is also increasing [4]. One of the most important complications that can be encountered in IBD patients is the development of malignancy. There are several reasons for the development of malignancy based on IBD [5]. One of the main causes of these is the presence of long-term chronic inflammation [1]. Long-term chronic inflammation triggers tumor formation. The malignancies that develop on this basis are mostly gastrointestinal system malignancies such as colorectal cancer, small intestine cancers, and cholangiocarcinoma [6]. However, exposure to immunosuppression is increasing in patients with the new treatments developed today. In conclusion, another important reason for the development of malignancy in IBD patients is prolonged immunosuppression [7]. Immunomodulators such as thiopurines and methotrexate and biological agents such as antitumor necrosis factors (anti-TNF) are the cause of this immunosuppression.

In fact, in some recent studies, it has been observed that new treatment strategies provide better IBD management, and therefore, the prevalence of IBD-induced malignancy has decreased, on the contrary, the development of immunosuppression-related malignancy has increased [7]. In addition, there is an increasing frequency of the development of extraintestinal system malignancies [8].

Except for IBD itself and immunosuppressive treatments, factors such as patient age, duration of disease, treatment exposure duration, diffusiveness of intestinal involvement, presence of primary sclerosing cholangitis (PSC), and presence of obesity are important factors in the development of malignancy [8, 9].

Gastrointestinal system malignancies, especially colorectal cancers, constitute most of the cancers that develop based on IBD [10, 11]. In our study, compatible with the literature, the most common cancer developing in IBD patients was colorectal cancer.

Many extraintestinal system cancers, such as head and neck cancers, lymphoma, skin cancers, genito-urinary system cancers, liver and biliary tract cancers, breast cancer, lung cancer, and thyroid cancer, have been reported in IBD patients [12, 13]. Liver and biliary tract cancers were observed more frequently, especially in patients with accompanying PSC [14]. In our study, two male UC patients had PSC, but no development of malignancy was observed in these patients. In the study by Samadder et al., examining the development of colorectal cancer in IBD patients, it was seen that the greatest risk factor for the development of colorectal cancer was the presence of PSC in IBD [15].

With the use of immunomodulatory and anti-TNF drugs in many areas, it is very important to examine the safety profile of these drugs. It is known that there is a risk of developing many malignancies, especially lymphoproliferative malignancies, especially with immunomodulatory drugs [16]. Although it is known that anti-TNF drugs have risks of malignancy due to their immunosuppressive effects, some studies have shown that this effect is less than that of immunomodulatory drugs [17]. In our study, no significant difference was found between the development of malignancy between immunomodulatory drugs and biological drugs. Another issue that increases the risk of malignancy development is the duration of use of the drugs as well as the drugs used. Cumulative immunomodulatory exposure increased the risk of cervical intraepithelial neoplasia in women, according to a study by Kreijne et al. [7], but in the study by Tassone et al., no association was seen between the cumulative duration of use of thiopurines, anti-TNF, or other biologic agents and the development of IBD and malignancy [18]. Likewise, in our study, no relationship was observed between the duration of immunosuppressive drug use and the development of malignancy.

Skin cancers are one of the cancers with the highest incidence of immunosuppressive therapies. Studies have shown that patients with immunomodulator and anti-TNF use increase the risk of malignancy, especially skin cancers [19].

In IBD, other than the disease itself and the drugs used in the treatment, other risk factors related to the risk of malignancy are patient-related factors. These include sex, patient age, comorbidities, family history, and body mass index [20]. Studies have shown that males have a higher risk of malignancy development in male IBD patients than females [20]. In our study, in survival analyses, a significant relationship was found between the female sex and the development of malignancy, in contradiction with the literature [3], but it should be noted that this significance is quite a borderline value. However, a recent study by Shani et al found that female sex was an increased risk factor for the development of second malignancies in IBD patients [21]. However, we are aware that this result also contradicts the general literature. The finding that female sex is associated with higher malignancy risk contradicts the general literature, but this is only a borderline result and lost significance in multivariate analysis in our study. We consider the results regarding female sex to be exploratory findings, not definitive. As seen in Table 3, when this data was examined in multivariate Cox regression analyses, it was seen that the difference in the development of malignancy in men and women was not supported. There was no difference between the male and female patients in our study regarding age, BMI, follow-up duration, and duration of immunosuppressive drug use. In addition, consistent with the literature [3], colorectal cancers were significantly more common in male patients.

In the study by Samadder et al, which examined the effect of having a family history of colorectal cancer on the development of colorectal cancer in IBD patients, it was observed that the risk of developing colorectal cancer increased 8-fold in IBD patients who had first-degree relatives with colorectal cancer. Unfortunately, we could not comment on this issue because we could not access the family history of the patients in our study [15].

Studies have shown that the development of malignancy increases as the age of the disease increases, especially in early-onset IBD [22], but in our study, the development of malignancy was found to be more significant in those diagnosed with IBD at an older age. The risk of colorectal cancer in IBD increases over time [23], but in our study, the development of malignancy was found to be more significant in those whose disease age was less than 8 and 10 years. The colorectal cancer rate in our study was 38.5% (n = 5), and the rate of patients developing malignancies other than colorectal cancer was higher (61.5%).

The risk of malignancy increases in elderly patients [24]. In our study, advanced age was found to be a risk factor for the development of malignancy in IBD in survival analysis.

It is known that obesity is an important risk factor for many malignancies. Studies examining the development of malignancy with one-to-one high BMI and IBD are limited. In our study, it was shown that high BMI is an important risk factor for the development of malignancy in IBD patients in both survival analysis and multivariate analysis.

In patients with IBD, the incidence of de-novo malignancy development and recurrence of malignancy in patients with a previous history of malignancy is higher than in patients without a history of malignancy [25]. Therefore, the use of immunosuppressive drugs in patients with a previous history of malignancy is a controversial issue for both physicians and patients. However, in some studies, no relationship was found between the use of conventional immunosuppressive drugs and the risk of recurrence or de novo cancer development in patients with a previous history of malignancy [26, 27].

Anti-TNF drug use is not recommended in patients with active malignancies, but there is little information on the use of anti-TNF drugs in patients with pre-existing malignancies. Studies have not found a direct relationship between anti-TNF drugs and the development of recurrence or de-novo cancer [28, 29].

In our study, no recurrence or new malignancy development was observed in any of the patients with pre-existing malignancy.

If malignancy develops during the follow-up of IBD patients, immunosuppressive drugs may be discontinued, drug-free follow-up according to the activity of the disease or, if necessary, the use of biological agents such as vedolizumab or ustekinumab, which are less risky in terms of malignancy, may be considered [30].

There are some limitations to this study. We think that the most important limitation is its retrospective nature. This caused the examination of only limited variables that could be obtained during the patient data collection. Other limitations are that the number of patients was relatively small for approximately 22 years of patient follow-up, and that the follow-up period of some patients was shorter than others. The main reason why the number of patients is relatively small compared to the follow-up period is that the was its retrospective nature. The other one is that this is a single-center study. We have presented our center data, and only 13 malignancy cases among 696 patients (1.86%) were reported. The small number of patients reaching the endpoint may affect the generalizability of survival and multivariate analysis results. Although it is not always possible to generalize the results of single-center studies, we believe that they can at least provide insight and guidance for subsequent studies. As this is a prevalence study, our primary objective was to draw attention to the occurrence of malignancies within the IBD population. The findings remain important in highlighting a potential clinical concern and serve as a basis for future research with larger cohorts and longer follow-up periods.

## 5. Conclusions

The prevalence of IBD is increasing in the world, and the management of the disease is improving with new treatment options. All of these lead to the prolongation of the disease duration and the many complications, including malignancies, over time. The development of malignancy is one of the most feared complications of IBD, and early diagnosis and correct management are very important. For this reason, patients with IBD should be followed carefully for the development of malignancy, and a multidisciplinary approach should be used in the management of these patients, if necessary. According to the results of our study, patients with high BMI should be followed more carefully in terms of malignancy development. It should be taken into account that the risk of colorectal cancer is higher in male patients, and it should not be forgotten that older patients are at greater risk according to survival analyses.

## Figures and Tables

**Table 1:** Characteristics of the patients

	All patients (n = 696)	Crohn’s Disease (n = 382)	Ulcerative colitis (n = 315)	*p*-value

Gender (n, %)				
Female	320 (45.9)	179 (46.9)	141 (44.8)	0.580
Male	376 (54.1)	203 (53.1)	174 (55.2)	

**Age (mean ± SD)**	33.4 ± 13.1	32 ± 12.5	35.1 ± 13.8	**0.002**

**BMI (kg/m** **^2^** **) (mean ± SD)**	23.2 ± 4.5	22.5 ± 4.6	24 ± 4.2	**0.006**

**Disease age (month) (mean ± SD)**	93.1 ± 64.8	87.9 ± 60.5	99.5 ± 69.1	**0.020**

Malignancy development after IBD diagnosis (n, %)	13 (1.9)	6 (1.6)	7 (2.2)	0.527

Malignancy history before IBD diagnosis (n, %)	10 (1.4)	5 (1.3)	5 (1.6)	0.761

**Abbreviations:** *BMI, Body mass index; IBD, Inflammatory bowel disease; SD, Standard deviation*

**Table 2:** Comparison of patients who developed and did not develop malignancy

	With malignancy (n = 13)	Without malignancy (n = 683)	*p*-value

**Gender (n, %)**			
Female	10 (76.9)	310 (45.4)	**0.046**
Male	3 (23.1)	374 (54.6)	

**Age (mean ± SD)**	42.8 ± 12.2	33.2 ± 13.1	**0.010**

**Age ≥ 40 years at diagnosis (n, %)**	10 (76.9)	193 (28.3)	**0.010**

**BMI (kg/m** **^2^** **) (mean ± SD)**	28.4 ± 5.3	23 ± 4.4	**0.037**

**BMI ≥ 25 kg/m** **^2^** ** at diagnosis (n, %)**	6 (46.2)	90 (13.2)	**0.002**

Disease age (month) (mean ± SD)	116.1 ± 76.8	92.7 ± 64.5	0.197

Duration of immunosuppressive drug (month) (mean ± SD)	97 ± 77.2	91.5 ± 62.3	0.788

IBD type (n, %)			
Crohn’s disease	6 (46.2)	376 (55)	0.820
Ulcerative colitis	7 (53.8)	308 (45)	

**Abbreviations:** *BMI, Body mass index; IBD, Inflammatory bowel disease; SD, Standard deviation*

**Table 3:** Multivariate Cox regression analysis for risk factors of malignancy

	HR	95% CI for HR		p-value

		Lower	Upper	
Gender (female)	1.634	0.361	7.403	0.524
Age (≥ 40 years old)	1.706	0.262	11.133	0.577
**BMI (≥ 25 kg/m** **^2^** **)**	13.991	1.307	149.749	**0.029**

*Table notes: BMI, Body mass index; CI, Confidence interval; HR, Hazard ratio;*

The model included gender, age at diagnosis of inflammatory bowel disease (≥ 40 or < 40 years old), and BMI (≥ 25 or < 25 kg/m^2^) which were found to be significantly related to malignancy in survival analyses.

**Table 4:** Characteristic of patients who developed malignancy

Gender	Age	Phenotype of IBD	Disease age at diagnosis of malignancy (months)	Malignancy	Medical treatment before diagnosis of malignancy	Management of malignancy	Management of IBD
**F**	66	UC	147	Colon adenocarcinoma	5-ASA	Total colectomy	5-ASA
**F**	74	CD	165	Kaposi’s sarcoma	AZA, 5-ASA	Follow up	5-ASA
**F**	78	UC	174	Pancreas adenocarcinoma	5-ASA	RT and PTC insertion	5-ASA
**M**	61	UC	207	Colon adenocarcinoma	5-ASA	Total colectomy	5-ASA
**F**	51	CD	9	Diffuse large B-cell lymphoma	5-ASA, MMF	CT	5-ASA
**F**	68	UC	168	Colon adenocarcinoma	5-ASA	Total colectomy	5-ASA
**F**	39	UC	97	Cervix cancer	5-ASA, AZA	Operation, CT, and RT	5-ASA
**F**	58	CD	68	Neuroendocrine tumor	5-ASA, AZA	Operation	5-ASA, AZA
**F**	38	UC	72	Spindle cell tumor	5-ASA, AZA	Operation	5-ASA, AZA
**F**	62	CD	223	Mycosis fungoides	5-ASA, AZA	PUVA therapy	5-ASA
**M**	45	CD	264	Rectum adenocarcinoma	5-ASA, AZA	Operation and CT	
**M**	62	UC	16	Sigmoid colon adenocarcinoma	Adalimumab	Anterior resection	5-ASA
**F**	55	CD	143	Breast cancer	Infiliximab	CT	Vedolizumab

**Abbreviations:** *5-ASA, 5-aminosalicylic acid; AZA, Azatiopurine; CD, Crohn’s disease; CT, Chemoterapy; F, Female; IBD, inflammatory bowel disease; M, Male; MMF, Micophenolat mofeti;l PTC, Percutaneous transhepatic cholangiography; PUVA, Psoralen Ultra-Violet A; RT, Radiotherapy; UC, Ulcerative colitis*
